# Retraction: miR-16 and fluoxetine both reverse autophagic and apoptotic change in chronic unpredictable mild stress model rats

**DOI:** 10.3389/fnins.2025.1642426

**Published:** 2025-06-13

**Authors:** Frontiers Editorial Office

The publisher retracts the article cited above.

Following publication, concerns were raised regarding the integrity of the images in the published figures. The authors failed to provide a satisfactory explanation during the investigation, which was conducted in accordance with Frontiers' policies.

This retraction was approved by the Chief Executive Editor of Frontiers. The authors received a communication regarding the retraction and had a chance to respond. This communication has been recorded by the publisher.

Frontiers would like to thank the users on PubPeer for bringing the published article to our attention.

